# The PBDE-209 Exposure during Pregnancy and Lactation Impairs Immune Function in Rats

**DOI:** 10.1155/2012/692467

**Published:** 2012-02-15

**Authors:** Xianbao Liu, Hong Zhan, Xu Zeng, Chunfang Zhang, Dunjin Chen

**Affiliations:** ^1^The Third Affiliated Hospital of Guangzhou Medical College, The Medical Center for Critical Pregnant Women in Guangzhou, Duobao Road, No. 63, Guangzhou 510150, China; ^2^Department of Pathology, Detroit Medical Center, Harper Hospital, School of Medicine, Wayne State University, 3990 John R, Detroit, MI 48201, USA

## Abstract

In the present study, we assessed the treatment effects of PBDE-209 administration on the immune function in rats during pregnancy and lactation. We harvested the blood and organs for flow cytometry, viability assay, enzyme-linked immunosorbent assay, and histological evaluation. The results of this study were the PBDE-209 exposure during pregnancy and lactation impairs immune function in rats. The results may contribute to understanding the mechanism of PBDE-209 in immune function.

## 1. Introduction

Polybrominated diphenyl ethers (PBDEs) are a group of bromide-containing compounds that has been wildly applied to many dairy products as flame retardants for several decades. The PBDEs have been proven to be life saving by allowing longer escape time and reduced overall damage during fire [1]. But there is really no evidence to support this claim that PBDEs have been used in house wiring, electrical appliances, TV, computers and mobile phones, building materials, and numerous plastic products [2–4]. While there are potentially 209 PBDE congeners, there are only 3 major commercial mixtures which contain a limited number, present in penta-, octa-, or decabrominated forms. PBDE-209 is not lipophilic—it accumulates in the blood and liver, bound to proteins. Many of the lower brominated congeners are lipophilic and can be accumulated in fatty tissue and pass into food chain. High concentration of PBDEs has been detected in tropical fish or lipid-rich oils. Therefore, PBDEs have become a constant environmental pollutant [5].

In recent years, increasing concerns have focused on the potential adverse effects of PBDEs in humans. Numerous studies have shown that PBDEs exert a wide range of toxic effects in many organ systems, such as disruption of thyroid function and neurotoxicity during development [6–8]. Further, mice exposed to isomer PBDE 47 suppress lymphocytes proliferation and antibodies production [9]. These findings suggest that exposure to PBDE may modulate the functions and structure of immune system. Among PBDEs, the polybrominated diphenyl ether-209 (PBDE-209, a deca-BDE) is a highly brominated PBDE with 10 bromine atoms and receives a lot of attentions. The PBDE-209 can be released into the environment by a variety of processes, such as emissions during manufacture of decaBDE-containing products or/and from the products themselves. The PBDE-209 has showed toxic effects during development of central nerve system in neonatal rats, especially in large doses [8]. Even though there have been multiple human studies on PBDEs, there is limited information regarding the adverse effects of PBDE-209 in the immune functions in animals and humans during pregnancy and lactation. Based on our previous findings that there is a decrease in immune function after the PBDE-209 exposure in vivo in pups [10], the present study is designed to further characterize the immune function impairment following PBDE-209 exposure in vivo during pregnancy and lactation in dams. Changes in immune function after PBDE-209 exposure in vivo is evaluated by measuring serum cytokines and immunoglobulin, as well as other specific biological markers and lymphocyte differentiation, and correlated with histopathological findings. We demonstrated a profound toxicity in immune system in rats during pregnancy and lactation following PBDE-209 exposure in vivo. Our findings indicate that exposure to PBDE-209 in vivo produces immunosuppressive effects in rats during pregnancy and lactation.

## 2. Materials and Methods

### 2.1. Animals

Female Sprague-Dawley rats, age of postnatal day (PND) 21, were purchased from the Experimental Animal Centre of Southern Medical University, China, then randomly divided into control and experimental groups. No significantly difference was observed between control and experimental group for their initial body weight. Rats were housed with 12-hour light/dark cycle in the pathogen-free unit and were provided with animal food and water. All behavioural studies were conducted between 2:00 PM to 4:00 PM. The rats were accustomed in SPF standards animal laboratory, and they were mated at 4:00 PM–7:00 AM. The animal care and experimental practice at Southern Medical University are regularly inspected by the University Animal Care and Use Committee and by governmental agencies to ensure compliance with international regulations and guidelines.

### 2.2. Chemicals

 PBDE-209 was obtained from Sigma-Aldrich Co. Ltd., USA. The purity of PBDE-209 was 98%. Mouse anti-rat FITC conjugated, anti-CD3 PE conjugated, anti-CD4 PE conjugated, anti-CD8 FITC conjugated, anti-CD161 and mouse PE-Cy5-labeled IgG1, FITC-labelled IgG1, and PE-labelled IgG1 were purchased from Serotech Co. Ltd., Netherlands. Primary mouse anti-rat IgG, goat anti-mouse immunoglobulins conjugated to horseradish peroxidase secondary antibodies, DAB Reagent set (KPL), serum IgM, IgG, interleukin-4 and interferon-*γ* ELISA kit were purchased from Jingmei Biotechnology Co. Ltd., China. Pentobarbital was purchased from Sigma-Aldrich, St. Louis, MO.

### 2.3. Experimental Groups

The rats were randomly divided into two groups: experimental and control. The experimental group (*n* = 20) were intragastrically administered PBDE-209 in 2 mL arachis oil (0.3 g/kg/day) from postnatal day 21 until their offsprings 3 weeks. The animals dosed from PND21 until their offspring were weaned were more than 14 weeks. The control group was given 2 mL purified arachis oil.

### 2.4. Experimental Protocol

After PBDE-209 administration, rats were anesthetised with intraperitoneal injection of pentobarbital (20 mg/kg) and scarified. The venous blood was collected in a 10 mL sterile tube from each rat without adding anticoagulant and stored at −20°C. Liver, spleen, thymus, and ovaries were dissected and weighed immediately after dissection. The dissected organs/tissues were fixed in 10% formalin for further histological processing. Prior to assay, blood samples were thawed out to room temperature by gentle swirling and inversion. All sera separated from blood at 3000 P/m for 15 minutes were stored at −20°C for further assay. All sera were assayed on the same day to avoid interassay variation.

### 2.5. Flow Cytometry

The lymphocytes were extracted as previously described [11]. Briefly, the thymus tissues were dissociated into a single cell suspension by passing through a 100/47 gauge nylon mesh (Tetko, Depew, NY). The lymphocytes are collected and cultured in 6-well plates with approximately 1 × 10^6^ cells/well. The cells were then centrifuged for 5 min at 1000 rpm and incubated with 10 *μ*L mouse anti-rat monoclonal antibodies in 300 *μ*L PBS as follows: FITC-conjugated monoclonal antibodies (Mab) against CD3, PE-conjugated anti-CD4, PE-conjugated anti-CD8. The cells were allowed to incubate without light source at 37°C for 20 min. After another 5 min centrifugation at 1000 rpm, the cells were resuspended with PBS and 300 *μ*L HBSS buffer solution and the lymphocytes were analysed by flow cytometry (excitation at 488 nm and emission at 525 nm, Becton Dickinson, USA) to detect T lymphocyte surface antigens.

### 2.6. Viability Assay by CCK-8

Previous experiment has showed that CCK-8 method was sensitive to detect lymphocyte cell viability at 5 *μ*g/mL phytohemagglutinin or lipopolysaccharide buffer with approximately 5 × 10^6^ T or B lymphocyte cells/mL. In present experiment, the aliquots were distributed into a 96-well plate and incubated for 72 h at 37°C. After the incubation period, CCK-8 solution was added to the culture medium and the cells were further incubated for 6 h. With adding DMSO, the absorbance at a wavelength of 450 nm with a referenced wavelength of 630 nm was measured.

### 2.7. Enzyme-Linked Immunosorbent Assay

 The concentrations of interferon-*γ*, IgM, and interleukin-4 in sera harvested from PBDE-209-treated rats were measured by enzyme-linked immunosorbent assay in accordance with previously described protocol. Briefly, 96-well microtiter plates (NUNC-Immunok Plate) were coated with immunoaffinity-purified polyclonal antibodies against the respective cytokines overnight at 4°C. After blocking the nonspecific binding with 1% albumin for 1 h, cytokine standards and experimental samples were loaded together and incubated for 90 min at 37°C. Biotin-conjugated antibody was added, followed by an additional incubation for 1 h at 37°C. Finally, 100 *μ*L of avidin-conjugated horseradish peroxidase (1 : 100 dilution; Jingmei, China) was added into each well. After 30 min incubation, the plates were washed and the colour reagent TMB (100 *μ*L/well) was added and allowed to react for 15 min, then reaction was stopped by 1 M H_2_SO_4_ and the OD was measured at 450 nm. The cytokine concentration was expressed as pg mL^−1^.

### 2.8. Histological Methods and Evaluation

For histological examination of organs, rats exposed to PBDE-209 were sacrificed on offspring postnatal day 21, and the liver, spleen, thymus, and ovaries were removed and placed in buffered formalin for a minimum of 24 h. After 10% formalin fixation, organs sampled for histology were dehydrated and paraffinized and embedded according to standard sampling and trimming procedures. 3 *μ*m sections were prepared and stained with haematoxylin and eosin within an automated machine. An observer who had no knowledge of the research intent and was blind to the origin of the sample performed the tissue analysis. The observer scored the severity of organs in the tissue sections. We made all evaluations on five fields per section and five sections per organ.

### 2.9. Statistical Analysis

All data were expressed as mean ± standard deviation (SD). Statistical analyses were performed using Statistical Package for the Social Sciences (SPSS) Version 13.0 for Windows. Comparisons of serum interferon-*γ* and interleukin-4, body and organ weights, T lymphocyte subset markers, lymphocyte viability, serum IgM, serum IgG, and NK cell surface markers between control and experimental groups were carried out using one-way analysis of variance (ANOVA) followed by a post hoc test (Bonferroni's method). We analyzed histological scored using the Kruskal-Wallis test followed by the Mann-Whitney *U*-test. A *P* value of less than 0.05 was considered to be statistically significant.

## 3. Results

### 3.1. Effects of PBDE-209 Exposure on Body and Organ Weight

The body weight of experimental group was significantly lighter than control group (*t* = 2.55, *P* < 0.05) (Table 1). Spleen and spleen index were heavier in experimental rats in comparison to control group (*t* = −4.90, −5.71, resp., *P* < 0.01). The thymus weight and thymus index of experimental rats were not significantly different from control group (*t *= 1.05, −0.98, resp., *P* > 0.05).

### 3.2. Effects of PBDE-209 Exposure on Surface Markers of T Cells or NK Cells

**Figure 1:**
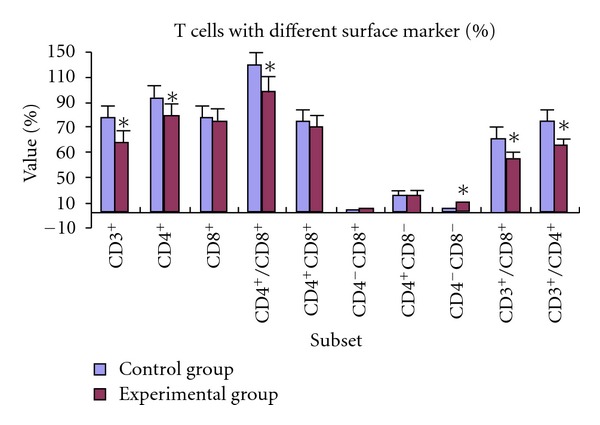
We measured the percentage of T lymphocytes in blood of exposed Sprague-Dawley (SD) rats using flow cytometric analysis. Values are expressed as mean ± standard deviation, and are representative of two separate experiments with twenty rats per group in each experiment. *P < 0.05 compared to control group.

Using flow cytometric analysis technique, the percentage of T lymphocytes in blood was measured in Sprague-Dawley rats exposed to PBDE-209 (Figure 1). The percentage of lymphocytes stained with CD3^+^, CD4^+^, CD3^+^CD8^+^, CD3^+^CD4^+^, and the ratio CD4^+^/CD8^+^ were decreased significantly in experimental group (*t* = 3.56, 5.09, 3.12, 4.0, and 3.77, resp.; *P* < 0.05). The percentage of CD4^−^CD8^−^ lymphocytes was significantly increased (*t* = −2.15, *P* < 0.05) in experimental group. No significant changes were observed in the percentage of CD8^+^, CD4^+^CD8^+^, CD4^−^CD8^+^, and CD4^+^CD8^−^ lymphocytes after exposure to PBDE-209 (*t* = 0.85, 0.98, −0.91, −0.08, resp., *P* > 0.05). There is significant decrease in surface CD161 expression on NK cells in experimental group when compared to control group (*t* = 9.27, *P* < 0.05) (Table 3).

### 3.3. Effects of PBDE-209 Exposure on Lymphocyte Viability

The CCK-8 method was applied in the present study to measure the viability of T and B lymphocytes from blood sample taken from PBDE-209-exposed Sprague-Dawley rats. The OD decreased in experimental group in applying with phytohemagglutinin stimulus, and increased in experimental group without phytohemagglutinin stimulus. OD decreased in experimental group in applying with lipopolysaccharide stimulus, decreased in experimental group without lipopolysaccharide stimulus. Stimulus index is stimulated index = OD value in stimulate hole/OD value in controlled hole. T lymphocyte stimulus index decreased in the experimental group in applying with phytohemagglutinin stimulus, B lymphocyte stimulus index increased in the experimental group in applying with lipopolysaccharide stimulus (Table 2).

### 3.4. Effects of PBDE-209 Exposure on Serum IgM, IgG, Interferon-*γ*, and Interleukin-4

Serum level of IgM, IgG, interferon-*γ*, and interleukin-4 was measured by enzyme-linked immunosorbent assay (ELISA). The serum levels of IgM of experimental rats were significantly lower than those in the control rats (*t* = 2.54, *P* < 0.05) (Table 3). Serum level of IgG was also significantly decreased in experimental group (*t* = 2.60, *P* < 0.05) (Table 3). No significant differences were detected in serum level of interleukin-4 or interferon-*γ* between experimental group and control group (*t* = 0.29, 1.29, resp., *P* > 0.05) (Table 3).

### 3.5. Histopathological Changes after PBDE-209 Exposure

**Figure 2:**
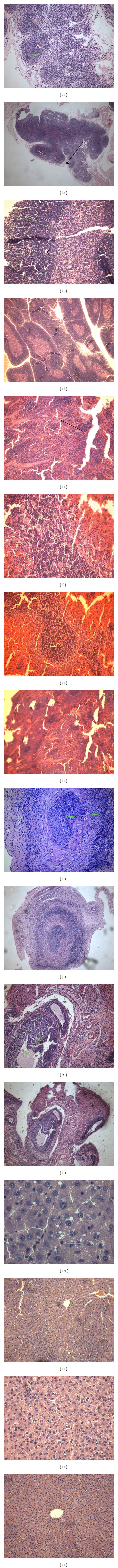
(a)–(o). Histological examination of microphotographs obtained from the tissues of twenty animals in each group revealed changes in the architecture of thymus, spleen, ovaries, and liver of treated (0.3 mg/kg/d) animals. Thymus: (a) experimental group (200X); connective tissues (arrow); (b) experimental group (100X)*；*connective tissues (arrow); (c) control group (200X), corticomedullary (green arrow) thymic corpuscles (black arrow); (d) control group (100X)*；* Spleen: (e) experimental group (200X); (f) experimental group (400X); (g) control group (200X), fibrous connective tissue(arrow); (h) control group (100X). Ovaries: (i) experimental group (200X), follicular atresia (arrow); (j) experimental group (100X); (k) control group (200X), follicular (arrow); (l) control group (100X)*；* Liver: (m) experimental group (200X), eosinophilic bodies (blue arrow), ballooned change (green arrow); (n) central vein (arrow) experimental group (100X); (o) control group (200X), eosinophil (arrow); (p) control group (100X).

The thymus, spleen, liver, and ovaries were sectioned, stained with H&E, and the slides were evaluated by pathologist. The experimental group showed an obvious change in morphology following PBDE-209 exposure (Figures 2(a)–2(p)). Histological scoring of thymus, spleen, ovaries, and liver injury increased in the experimental group (Table 4). The morphologic changes in thymus in experimental group include the following: thickened thymus capsule, decreased lymphoid tissue in cortex with adipose tissue replacement, and increased the size of medulla. The corticomedullary junction was obscure. Plenty reticular epithelial cells but scarce corpuscles are seen.

 The sections of spleen in experimental group showed a decrease in size and number of lymphoid nodules, thinner lymphatic sheath around arteries with less lymphocytes. In other hand, marked increase in fibrotic tissue with many macrophages is observed in spleen medulla.

The liver structure and the central vein structure in experimental group were changed dramatically. The hepatocyte sinusoids were compressed by the swelling hepatocytes with balloon degeneration. Eosinophilic changes and eosinophilic bodies were identified in hepatocytes.

Ovarian from experimental group showed atrophic changes with decrease in number of follicles and increase amount of fibrotic tissue.

## 4. Discussion

The PBDE-209 is a very popular brominated flame retardant. However, adverse effects of PBDE-209 use on public health, especially its immunotoxicity, remain obscure due to a paucity of toxicological and epidemiological data regarding long-term PBDE-209 exposure. A large body of studies indicate that PBDE-209 can be sustained in environment and accumulated in terrestrial biota, causing liver and thyroid damage and retardation of nerve system [12, 13]. Immunotoxicity of PBDEs has been previously reported in the Great Lakes ecosystem [14, 15]. However, immunotoxicity caused by PBDE-209 in dams following long-term exposure has not yet been reported. To our best knowledge, this is the first paper regarding immunotoxicity in dams after long term PBDE-209 exposure. After long-term exposure to PBDE or DE71, a mixture of PBDE, no change in body weight gain in perinatal or adult rat has been reported. In addition, no damage to human body is found below toxic levels of PBDEs [16–18]. In present study, dams exposed to PBDE-209 for more than 14 weeks (0.3 g/kg/day) showed decrease in body and spleen weight (Table 1).

In previous study, we demonstrated that maternal PBDE-209 exposure during pregnancy decreases the ability of learning and memory in mice. After long-term exposure to PBDE-209, determination of the level of Th1/Th2 polarising cytokines, such as interleukin-4 and interferon-*γ*, is very important to evaluate the immunotoxicity of PBDE-209 since the initial encounter with the bacteria, parasites, yeasts, and viruses is crucial for the developing harmful autoimmune response postnatal in two aspects [19]. On one hand, a Th1 is responsible for producing of complement-fixing IgG2a isotype antibodies and the activation of natural killer (NK) cells as well as cytotoxic CD8^+^ T cells that is releasing interferon-*γ* and perforin. On other hand, the Th2 cells produce the cytokine interleukin-4 and can activate mast cells and eosinophils to eradicate helminths and other extracellular parasites. Together, Th1- and Th2-specific cytokines promote growth and differentiation of a specific T-cell subset. In the present study, we observed no significant differences in levels of interferon-*γ* and interleukin-4 in rats exposed to PBDE-209, although Watanabe reported that a significant elevation in level of interferon-*γ* in animal treated with 1000 ppm DBDE [20]. The possible underlying causes for this may be related to the duration of PBDE-209 exposure or the dosing of PBDE-209 used in present study that was not sufficient to induce these effects.

T lymphocytes play a pivotal role in immune defence. Therefore, T lymphocytes are good candidates for studying immunotoxicity and risk assessment. T lymphocytes play roles in cellular immune regulation and immune surveillance. A variety of subsets of T lymphocytes can be identified by their cell surface markers, such as CD3 and CD4. B lymphocytes involve humoral immune regulation. The third type of immune cells, the NK cells, participates in cellular immune regulation and immune surveillance. We demonstrated a decreased proportion of CD3^+^, CD4^+^, CD3^+^CD8^+^, CD3^+^CD4^+^, CD161^+^, and CD4^+^/CD8^+^ T cells (Figure 1) in the thymus of PBDE-209-exposed dams. The percentage of CD161^+^ NK cells (Table 3) was also decreased. However, the proportions of CD8^+^, CD4^+^CD8^+^, CD4^−^CD8^+^ and CD4^+^CD8^−^ were changed following PBDE-209 treatment. We measured the proliferation of T and B lymphocytes (Table 2) from exposed rats by using CCK-8 method. The CCK-8 method is similar to the MTT method in technique. Previous experiments have showed that CCK-8 method was more sensitive than other methods in detecting lymphocyte proliferation in response to 5 *μ*g/mL phytohemagglutinin or lipopolysaccharide. By CCK-8, the T and B lymphocyte multiplication was suppressed after PBDE-209 treatment. Interactions between innate immune system and adaptive immune reactions are now widely viewed as essential for a normal immune response [21]. Most pathogens elicit a humoral immune response that is characterised by an early rise in antigen-specific IgM followed by affinity maturation, isotype switching, and the ensuing rise in antigen-specific IgG, IgA, and IgE antibodies. Therefore, we measured change in level of IgM and IgG in PBDE-treated rats in present study. The levels of serum IgG and IgM (Table 3) were significantly decreased. This finding suggests that the interaction between innate immune mechanisms and adaptive immune reactions could be interrupted in PBDE-209-exposed dams.

Perinatal PBDE exposure has been shown histopathologically to exacerbate pneumonia in the lungs of offspring mice given RSV infection. As Watanabe et al. reported, histopathological analysis of lungs of several offspring mice born to dams exposed to PBDE at 100 ppm, typical features of pneumonia with RSV infection, including hypertrophy of the bronchial epithelium and infiltration of lymphocytes, were apparent [20]. In a follow-up study, Leo et al. have reported that exposure to a commercial penta-PBDE mixture, PBDE-71, caused obvious histopathological changes in the adrenal glands, liver, and ileum [22]. In present histopathological analysis, our findings indicate that PBDE-209 exposure induced histopathological changes in thymus, spleen, liver and ovaries due to PBDE-209 exposure (Figures 2(a)–2(p)). Thickened thymus capsule decreases lymphoid tissue in cortex with adipose tissue replacement and increases the size of medulla in thymus. The corticomedullary junction was obscure. Plenty reticular epithelial cells but scarce corpuscles are seen. The sections of spleen showed a decrease in size and number of lymphoid nodules, thinner lymphatic sheath around arteries with less lymphocytes. In other hand, marked increase in fibrotic tissue with many macrophages is observed in spleen medulla. The liver structure and the central vein structure were changed dramatically. The hepatocyte sinusoids were compressed by the swelling hepatocytes with balloon degeneration. Eosinophilic changes and eosinophilic bodies were identified in hepatocytes. Atrophic changes with decrease in number of follicles and increase amount of fibrotic tissue in ovarian.

In conclusion, the findings in present study demonstrate, for the first time, that PBDE-209 exposure induced immunotoxicity. Exposure to large doses of PBDE-209 not only inhibits immune function but also alters the structure of immune organs. Rats exposure to PBDE-209 showed a suppression of lymphocyte proliferation and antibody production. The evidence regarding immunotoxicity induced by PBDE-209 in animal from present study may be useful for dealing with the potential risk of PBDE-209 exposure in public health. These findings also emphasize the importance of avoiding immune toxic contaminants in environment, such as PBDEs, particularly PBDE-209 in pregnant women. As such, the mechanism of immunotoxicity of PBDE-209 in present study should be elucidated as soon as possible.

## Figures and Tables

**Table 1:** Weight of body and organs (g).

Detection index	Control group	Experimental group
rat weight	284.00 ± 23.98	251.24 ± 32.73*
thymic weight	0.21 ± 0.03	0.20 ± 0.03
thymus index number	0.75 ± 0.10	0.80 ± 0.11
spleen weight	0.67 ± 0.04	0.75 ± 0.03**
spleen index number	2.37 ± 0.19	3.00 ± 0.30**

Mean ± standard deviation, *P < 0.05, **P < 0.01.

**Table 2:** T and B lymphocytes OD value.

Group	OD with PHA (LPS) stimulus	OD with no PHA (LPS) stimulus	OD stimulus index
Control group	0.87 ± 0.11	0.26 ± 0.09	3.55 ± 0.97
	(1.29 ± 0.48)	(1.03 ± 0.48)	(1.21 ± 0.04)
Experimental group	0.70 ± 0.17*	0.42 ± 0.10**	1.69 ± 0.20**
	(0.57 ± 0.11**)	(0.31 ± 0.12**)	(1.80 ± 0.33*)

Mean ± standard deviation, *P < 0.05, **P < 0.01.

**Table 3:** IgM, IgG, IFN-*γ*, and IL-4 levels in serum as measured by enzyme-linked immunosorbent assay and percentage of NK cells with CD161 surface markers.

Group	IgM (*μ*g/mL)	IgG (*μ*g/mL)	IFN-*γ* (pg/mL)	IL-4 (pg/mL)	CD161^+^ (%)
Control group	6.06 ± 1.51	17.02 ± 0.67	22.57 ± 20.47	16.07 ± 4.17	12.05 ± 0.88
Experimental group	4.36 ± 1.49*	16.31 ± 0.45*	12.27 ± 14.96	15.53 ± 3.63	6.23 ± 1.33**

Mean ± standard deviation, *P < 0.05, **P < 0.01.

**Table 4:** Histological scoring of thymus, spleen, ovaries, and liver injury.

Group	Thymus	Spleen	Ovaries	Liver
Control group	ND	ND	ND	ND
Experimental group	2.5 ± 0.20**	2.3 ± 0.15**	3.1 ± 0.16**	3.5 ± 0.13**

ND: not detectable, mean ± standard deviation, Mean ± standard deviation, *P < 0.05, **P < 0.01.
